# GalNAc Carbohydrate Prevents the Formation of Neutrophil Extracellular Traps and Increase Myeloperoxidase Enzyme Activity in Interactions of Neutrophils and *Entamoeba histolytica* Preincubated With GalNAc

**DOI:** 10.1155/bmri/8280585

**Published:** 2026-04-10

**Authors:** David Levaro-Loquio, Andrea Cruz-Baquero, Germán Higuera-Martínez, José De Jesús Serrano-Luna, Arturo Contis-Montes de Oca, Edgar Abarca-Rojano, Ivonne Maciel Arciniega-Martínez, Aldo Arturo Reséndiz-Albor, Judith Pacheco-Yépez

**Affiliations:** ^1^ Sección de Estudios de Posgrado e Investigación, Escuela Superior de Medicina, Plan de San Luis S/N, Casco de Santo Tomas, Instituto Politécnico Nacional, Ciudad de México, Mexico, ipn.mx; ^2^ Bacteriología, Facultad de Ciencias de la Salud, Universidad Colegio Mayor de Cundinamarca, Bogotá, Colombia, unicolmayor.edu.co; ^3^ Departamento de Biología Celular, Centro de Investigación y de Estudios Avanzados del IPN (CINVESTAV), Ciudad de México, Mexico

## Abstract

During amoebic infection, neutrophils are the first cells of the immune system to interact with trophozoites of *Entamoeba histolytica*, initiating amoebicidal activity. Different lectins and carbohydrates can participate in the mechanism of adhesion between *E. histolytica* and the target cell through the recognizing of carbohydrates by the lectins present in the cells and the parasite. The formation and release of NETs (neutrophil extracellular traps) are damage mechanisms in response to amoebae. NETs consist of DNA and antimicrobial enzymes such as myeloperoxidase (MPO). It is not known whether carbohydrates are able to block the formation and release of NETs or their compounds, such as MPO enzyme, in the interactions between carbohydrates–preincubated amoebae and neutrophils. *E. histolytica* 260 kDa and 220‐kDa lectins are glycoproteins that can stimulate the formation of neutrophil NETs. Our results showed that the formation of neutrophil NETs was prevented mainly by the carbohydrates N‐acetyl‐*α*‐D‐galactosamine (GalNAc) or N‐acetyl‐*α*‐D‐glucosamine (GlcNAc) in the interactions of neutrophils with carbohydrate preincubated *E. histolytica* but did not prevent a significant increase in the activity of the MPO enzyme or damage to trophozoites, suggesting that other mechanisms of neutrophil damage, such as MPO enzyme, are involved in amoebic damage. Our results revealed that incubation of amoebae with GalNAc increased neutrophil MPO enzyme activity, possibly in part through the inhibition of the amoebic 260‐kDa lectin.

## 1. Introduction

*Entamoeba histolytica* was identified by Schaudinn in 1903 and its name derives from its ability to cause tissue lysis [1]. Amoebiasis, caused by *E. histolytica*, remains an important public health problem, especially in developing countries. It is estimated that approximately 10% of the world′s population is affected by this parasitic infection. The main route of transmission is the consumption of food or water contaminated with *E. histolytica* cysts [2, 3]. Amoeba primarily infects the gastrointestinal tract and cause colitis. In certain cases, it invades the intestinal wall and spreads hematogenously to extraintestinal sites, with a predilection for the liver, where it causes amoebic liver abscess (ALA) [4]. *E. histolytica* has been described as causing lesions in the hepatic parenchyma, highlighting an exacerbated inflammatory response composed of polymorphonuclear leukocytes (PMNs), mainly neutrophils during the acute phase, and macrophages during the chronic phase in hamster model, which is known to be susceptible [4].

Several virulence and pathogenic factors have been characterized in *E. histolytica*, including 260 kDa and 220‐kDa lectins [5]. Parasite adhesion occurs mainly through the 260‐kDa surface lectin (Gal/GalNAc lectin), which binds to exposed terminal Gal/GalNAc residues on glycoproteins present on target cells. The Gal/GalNAc–inhibitable lectin of *E. histolytica* has been shown to be critical for in vitro amoebic adherence and for cytolysis of mammalian tissue culture cells and human PMNs [6–8].

Other works have also reported the isolation and purification of a protein with lectin‐like properties from *E. histolytica* trophozoites [9]. This lectin was obtained by electroelution, and electrophoretic analysis revealed a band with a molecular weight of 220 kDa. This 220‐kDa lectin agglutinates human erythrocytes and recognizes polymers of GlcNAc, chitin, and hyaluronic acid; and is also related to the adherence of the parasite to the target cells [9].

Studies have shown that activated neutrophils destroy in vitro *E. histolytica* via oxidative and nonoxidative mechanisms. Neutrophil release decondensed chromatin called NETs. NETs are mainly composed of DNA, histones, and other antimicrobial components (e.g., bacterial permeability‐enhancing protein, MPO enzyme, elastase, and lactoferrin among others) [10, 11]. NETs can bind and destroy several protozoa, such as *Leishmania amazonensis, Plasmodium falciparum*, and *Toxoplasma gondii* [12–14]. The formation of NETs plays an important role in the in vitro destruction of *E. histolytica* trophozoites because their large size prevents them from being phagocytosed by neutrophils [15]. Neutrophils contain MPO, which is a cationic enzyme located in the primary azurophil granules of neutrophils and immature monocytes. When released by neutrophils, MPO binds to monocytes and can produce ROS and induce the release of proinflammatory cytokines (i.e., TNF‐*α*, IL‐1, IL‐6, and IL‐8). Active MPO enzyme utilizes H_2_O_2_ produced by neutrophils to oxidize chloride ions and produce the highly cytotoxic hypochlorous acid (HOCl). MPO is stored in the azurophilic granules of naive neutrophils [16]. In addition, MPO is important for the formation and function of NETs [17]. There is no information on the effects of GalNAc and GlcNAc carbohydrates on NETs formation when host cells interact with amoebae preincubated with carbohydrate. The present study explored the NETs formation and MPO enzyme activity, as well as adhesion events during interactions between mouse neutrophils and *E. histolytica* preincubated with GalNAc and GlcNAc carbohydrates.

## 2. Material and Methods

### 2.1. Amoebic Cultures

*E. histolytica* trophozoites of the HM‐1: IMSS strain were cultured axenically at 37°C in Diamond′s trypticase yeast iron extract (TYI‐S‐33) medium supplemented with 10% bovine serum (Gibco, New York, United States, 26140079). The trophozoites were harvested at the end of the logarithmic growth phase (48 h) by chilling to 4°C. The trophozoites were concentrated by centrifugation at 300 × g for 5 min. The pellet was resuspended, amoeba were counted and used immediately [18].

### 2.2. Experimental Animals

Male 9‐week‐old BALB/c mice weighing 30 g were used for neutrophil isolation (*n* = 35). All animal procedures followed the federal regulations in Mexico regarding the use and care of laboratory animals (NOM‐062‐ZOO‐1999; Ministry of Agriculture, México City, México). The Escuela Superior de Medicina′s Institutional Animal Care and Use Committee acted as the regulatory office for approving research protocols and approved the animal care and handling of the mice (Protocol Number ESM‐CICUAL 07/26‐08‐2019).

### 2.3. Neutrophil Purification

Neutrophils were isolated from cardiac blood taken from mice, using heparin Vacutainer tubes after the animals were anesthetized with 1.3 mg of sodium pentobarbital (0.5 mg/100 g) intraperitoneally (Pisa, CDMX, MEX). The isolation of the neutrophil‐enriched fraction was performed by density gradient centrifugation using Histopaque 1077 (Sigma‐Aldrich, Missouri, United States, 1077) and 1119 (Sigma‐Aldrich, Missouri, United States, 11191). Briefly, 4 mL of Histopaque 1077 was placed over 4 mL of Histopaque 1119, and then, 6 mL of the collected blood was diluted 1:2 with Hank′s balanced salt solution (HBSS) (Invitrogen Life technologies, New York, United States, 24020117) and poured over this discontinuous density gradient. The tube was centrifuged at 758 × g for 30 min at 20°C. At the end of centrifugation, the neutrophil layer was collected and then washed with HBSS. A Neubauer chamber and an optical microscope (Seiwa optical microscope, Japan) (40×) were used for viability evaluation in a trypan blue assay (Sigma‐Aldrich, Missouri, United States, 15250061) [19, 20].

### 2.4. Interaction of Mouse Neutrophils With *E. histolytica* Trophozoites Preincubated With Carbohydrates

Before *E. histolytica* trophozoites interact with neutrophils, the amoebic lectins are blocked with different carbohydrates. For this purpose, *E. histolytica* trophozoites were preincubated for 20 min with each carbohydrate, namely GalNAc (Sigma‐Aldrich, Missouri, United States A8625A2795), GlcNAc (Sigma‐Aldrich, Missouri, United States A8625), or Mannose (Manno) (Sigma‐Aldrich, Missouri, United States, 1375182), at a concentration of 50 mM. The neutrophils (1 × 10^6^ cells/mL) were plated onto glass coverslips in a 24‐well plated and interacted with the carbohydrate pretreated *E. histolytica* trophozoites (5 × 10^4^ cells/mL) for 20, 40, 60, and 90 min of interaction at 37°C [19].

### 2.5. Detection of NETs in BALB/c Mouse Neutrophil Interactions With *E. histolytica* Preincubated With Carbohydrates by Fluorescence Microscopy

For the visualization of NETs, trophozoites were preincubated with carbohydrates and then interacted with neutrophils. After interaction, the samples were fixed with 4% paraformaldehyde (Sigma‐Aldrich, St. Louis, Missouri, United States, 158127) for 30 min at 37°C, and the samples were washed with PBS‐Tween. The cells were permeabilized with 0.5% Triton X‐100 (Invitrogen, New York, United States, HFH10) for 20 min and blocked with 1% BSA (Invitrogen, New York, United States, 15561020) for 30 min at 37°C. Subsequently, 200 *μ*L of anti‐*E. histolytica* polyclonal rabbit IgG diluted 1:3000 was incubated overnight, after which it was detected using a secondary donkey antirabbit IgG (H + L) Alexa Fluor‐647 antibody (Abcam, CAM, United Kingdom, ab150075) diluted 1:5000 and incubated for 4 h. Anti‐MPO polyclonal rabbit IgG conjugated to Alexa Fluor‐350 (Bioss Antibodies, Massachusetts, United States, BS‐4943R‐A350) as primary antibodies (1:1000 dilution in PBS‐T) was used and incubated for 4 h. The samples were counterstained with SYTOX Green (Thermo Scientific, Massachusetts, United States, S7020) to observe the DNA according to the manufacturer′s instructions. Finally, the slides were washed in PBS and mounted with VECTASHIELD (Vector Laboratories, California, United States, H‐1900) mounting medium [19]. Images were collected and analyzed with an Axioskop 2 MOT Plus confocal fluorescence microscope at 60× (Carl Zeiss, MX).

The colocalization analysis of fluorescence images, see Figures S1, S2, S3, and S4, were as follows: Three ROIs (336 × 336 pixels) were randomly selected from the green or blue‐channel interaction with red in the merged images (test set). The analyzed images of ROIs were merged with the different‐channel images by Fiji to observe the colocalization. The correlation coefficient (Pearson) between the colocalization intensity of DNA or MPO on trophozoites was calculated. In ideal colocalization, the R total should be equal to 1 [21].

### 2.6. DNA Quantification and MPO Enzyme Activity

The supernatants were collected from the neutrophil–trophozoite interaction. Prior to collecting the supernatants, NETs buffer (0.1‐g BSA+10 *μ*L of NaCl in 10 mL of RPMI at 37°C) was prepared. The interactions were carried out in a 2.0 mL tube; samples were centrifuged at 200 × g for 5 min at 22°C and the supernatant was recovered. After removing the supernatants, 500 *μ*L of NETs buffer and 10 *μ*L of plus Nuclease S7 enzyme (500 mU/mL) (Roche, BAS, SWI, 10107921001) were added to the pellet to release NETs; the tubes were incubated for 15 min at 37°C, the reaction was stopped with 10 *μ*L of EDTA (5 mmol/L), centrifuged at 15,000 × g for 5 min at 22°C, and the supernatant associated with NETs was subsequently obtained. The DNA in the supernatant associated to NETs was quantified using a Quant‐iT PicoGreen dsDNA kit (Invitrogen Life technologies, New York, United States, P7589) according to the manufacturer′s instructions. PMA was used as a control for the NETs induction measurements; additionally, DNAse (Roche, BAS, SWI, 11284932001) (4 U/mL) was used as a negative control [19].

The activity of the MPO enzyme was quantified using the first supernatant that was collected. Fifty microliters of the supernatant were added to a 96‐well plate, followed by an equal volume (50 *μ*L) of TMB (3,3 ^′^,5,5,5 ^′^‐tetramethylbenzidine 98%) (Sigma‐Aldrich, Missouri, United States, T2885). After that, the reaction started by adding 50 *μ*L of H_2_O_2_ (5 mM/L in water, high purity) (Sigma‐Aldrich, Missouri, United States, 31642). After a 10‐min incubation, the mixture was observed for color change. The reaction was stopped by adding 50 *μ*L of H_2_SO_4_ (2.5 mol/L) (Sigma‐Aldrich, Missouri, United States, 339741) [22]. The plates were centrifuged at 600 × g for 10 min, and 200 *μ*L of the supernatant from each well was transferred to a new plate. Optical density (OD) was measured at 405 nm using a Synergy HT microplate reader (Biotek, Vermont, United States). As a positive control, a group treated with phorbol 12‐myristate 13‐acetate (PMA) (20 nM) (Abcam, Cambridge, United States, ab120297) was used. As a negative control, the MPO inhibitor ABAH (4‐aminobenzoic acid hydrazide) (100 *μ*mol/L) (Sigma, Missouri, United States, A41909) was used [19, 20].

### 2.7. Studies of Carbohydrate Inhibition of Amoebic Adherence to Neutrophils (Rosette Formation)

Adhesion studies were conducted using neutrophils incubated with pretreated amoebae. The samples were fixed at the end of each interaction time. The interactions (triplicate) were carried out as previously mentioned. Rosette formation is defined as *E. histolytica* trophozoites associated with at least three neutrophils. Rosettes were quantified by counting 10 fields from three independent assays. The average number of rosettes per field was calculated using a Newbauer chamber. Brightfield images were acquired using an optical microscope (Nikon, Eclipse Ci‐S, Tokyo, Japan) [6–8].

### 2.8. Statistical Analysis

The data were analyzed using the GraphPad Prism Version 8.0. The experiments were performed in three separate biological triplicates, and each of these was measured three times technically. Statistical comparisons were performed using one‐way analysis of variance (ANOVA) or two‐way ANOVA, depending on the situation. The Bonferroni correction was used to adjust for multiple comparisons. When a significant main effect or interaction was found (*p* < 0.05), pairwise comparisons of group means were performed. These comparisons used the Bonferroni correction [23].

## 3. Results

### 3.1. NETs Release in the Interactions of Neutrophils and *E*. *histolytica* in Absence of Carbohydrates

**Figure 1:**
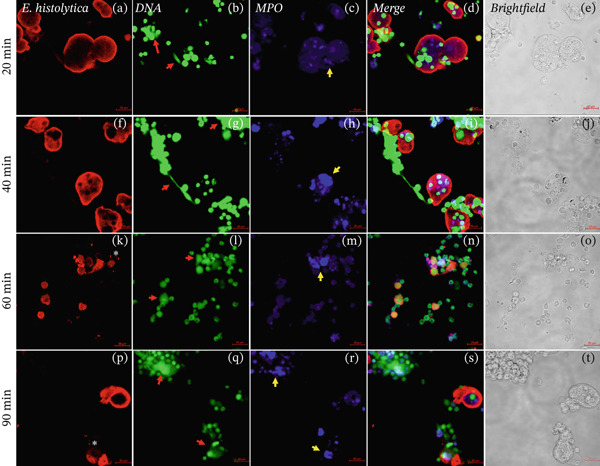
Neutrophil–amoeba interactions without preincubation with carbohydrates. The presence of NETs and MPO enzyme in the interactions of mouse neutrophils and E. histolytica (20, 40, 60, and 90 min). The trophozoites were stained with anti‐E. histolytica rabbit IgG (Panels a, f, k, and p; red), and the DNA was visualized by staining with SYTOX Green (Panels b, g, l, and q; green) and MPO with an Alexa 647–conjugated antirabbit antibody (Panels c, h, m, and r; blue), experiments were performed by triplicate. We observed the presence of NETs (red arrows), MPO enzyme (yellow arrows), and amoebic debris (asterisks). Additionally, the overlay of images (Merged Panels d, i, n, and s) and the brightfield images are observed (Panels e, j, o, and t). Bar: 20 μm.

The detection of NETs in interactions of mouse neutrophils with amoebae was evaluated by confocal microscopy using an anti‐*E. histolytica* antibody (red), an anti‐MPO antibody (blue), and SYTOX Green to stain DNA (green). We always observed the presence of DNA (Figures 1b, 1g, 1l, and 1q; red arrows) and MPO enzyme (Figure 1. (Figures 1c, 1h, 1m, and 1r; yellow arrows) in the neutrophil–amoebae interactions without preincubation with carbohydrates; NETs were observed in contact with trophozoites (Figures 1d, 1i, 1n, and 1s), but most trophozoites maintained their integrity at 20 and 40 min of interaction (Figure 1a,f). At 60 and 90 min, we observed neutrophils in contact with amoebic debris (Figure 1n,s), whereas MPO (Figure 1m,r) and NETs were observed upon contact with amoebic debris (Figure 1n,s). When evaluating the colocalization of NETs and MPO on *E. histolytica* trophozoites, it is observed that MPO begins to appear after 20 min of interaction, covering the trophozoites with a correlation of 0.72, whereas DNA is low at 20 min but increases during the first hour of interaction and can maintain a correlation of 0.61 with the trophozoite (Figure S1).

### 3.2. NETs Presence in the Interactions of Neutrophils and Amoebae Preincubated With Manno Carbohydrate

**Figure 2:**
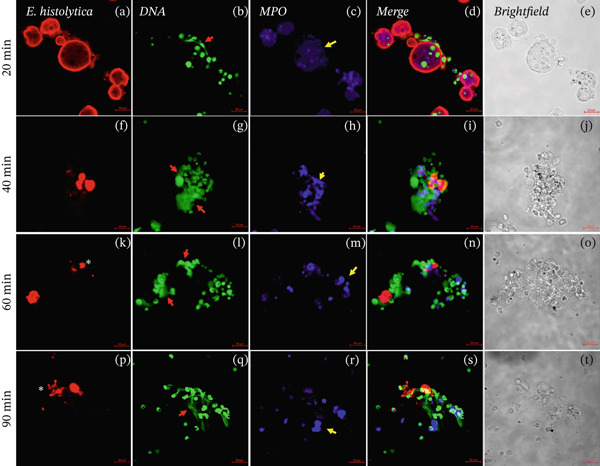
Interactions of neutrophils with amoebae preincubated with mannose. The presence of NETs and MPO enzyme in the interactions of mouse neutrophils and amoebae preincubated with Manno (50 mM) (20, 40, 60, and 90 min). The trophozoites were stained with anti‐E. histolytica rabbit IgG (Panels a, f, k, and p; red), DNA was visualized by staining with SYTOX Green (Panels b, g, l, and q; green) and MPO enzyme was visualized with Alexa 647–conjugated antirabbit antibody (Panels c, h, m, and r; blue), experiments were performed by triplicate. We observed the presence of NETs (red arrows), MPO (yellow arrows), and amoebic debris (asterisks). Additionally, the overlay of images (Merged Panels d, i, n, and s), and the brightfield images are observed (Panels e, j, o, and t). Bar: 20 μm.

NETs release in the interactions of neutrophils with amoebae preincubated with Manno at a concentration of 50 mM for 20, 40, 60 and 90 min; we observed the presence of DNA (Figures 2b, 2g, 2l, and 2q) and MPO enzyme (Figures 2c, 2h, 2m, and 2r; yellow arrows) at all times tested. NETs were observed in contact with trophozoites (Figure 2d,i) and amoebic debris (Figure 2n,s). At 60 and 90 min of interaction, amoebic debris (Figure 2k,p; asterisk), NETs (Figure 2l,q; red arrows) and MPO enzyme (Figure 2m,r; yellow arrows) were observed. Colocalization analyses detail the presence of NETs in greater interaction between 40 and 90 min with a correlation of 0.45, whereas MPO is shown from 20 min of colocalized interaction on amoebae with a correlation of 0.68 and remains during incubation times, decreasing in relation to the presence of amoebic detritus with a correlation of 0.48 (Figure S2).

### 3.3. Low Presence of NETs in the Interactions of Neutrophils and *E*. *histolytica* Preincubated With GalNAc Carbohydrate

**Figure 3:**
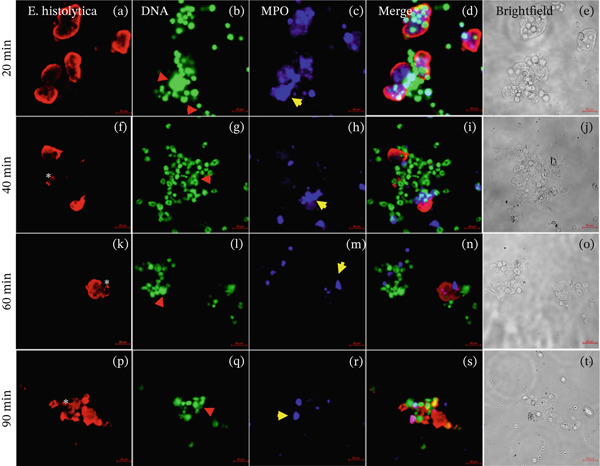
Interactions of neutrophils with amoebae preincubated with GalNAc. The presence of NETs and MPO enzyme in the interactions of mouse neutrophils and E. histolytica preincubated with GalNAc (50 mM) (20, 40, 60 and 90 min). The trophozoites were stained with anti‐E. histolytica rabbit IgG (Panels a, f, k, and p; red), DNA was visualized by staining with SYTOX Green (Panels b, g, l, and q; green) and MPO enzyme was visualized with Alexa 647–conjugated antirabbit antibody (Panels c, h, m, and r; blue), experiments were performed by triplicate. We observed the presence of NETs (red arrows), MPO enzyme (yellow arrows), and amoebic debris (asterisks). Additionally, the overlay of images (Merged Panels d, i, n, and s), and the brightfield images are observed (Panels e, j, o, and t). Bar: 20 μm.

NETs release in the interactions of neutrophils with amoebae preincubated with GalNAc at concentrations of 50 mM at 20, 40, 60, and 90 min; we observed a scarce presence of NETs in contact with trophozoites (Figures 3d, 3i, 3n, and 3s), and amoebic debris were observed (Figure 3k,p; asterisk). The presence of NETs was less evident (Figures 3g, 3l, and 3q; red arrows) with amoebae preincubated with GalNAc compared with the other groups at the same time of interaction. The presence of the MPO enzyme was evident with this carbohydrate at all interaction times (Figures 3c, 3h, 3m, and 3r; yellow arrows). When correlating the colocalization of NETs and MPO with *E. histolytica* trophozoites, it is observed that at 20 min, NETs show a correlation of 0.32, which decreases as interaction times increase to a correlation of 0.07, whereas MPO maintains a similar behavior with correlations ranging from 0.6 to 0.41. (Figure S3).

### 3.4. Presence of NETs in the Interactions of Neutrophils and *E*. *histolytica* Preincubated With GlcNAc Carbohydrate

**Figure 4:**
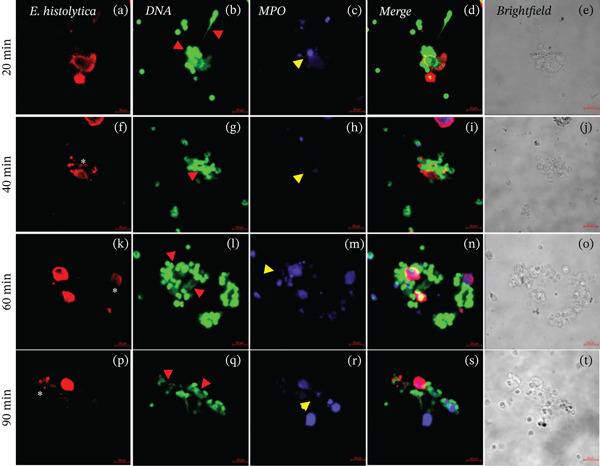
Interactions of neutrophils and amoebae preincubated with GlcNAc. The presence of NETs and MPO enzyme in the interactions of mouse neutrophils and amoebae preincubated with GlcNAc (50 mM) (20, 40, 60, and 90 min). The trophozoites were stained with anti‐E. histolytica rabbit IgG (Panels a, f, k, and p; red), DNA was visualized by staining with SYTOX Green (Panels b, g, l, and q; green), and the MPO enzyme was visualized with Alexa 647–conjugated antirabbit (Panels c, h, m, and r; blue), experiments were performed by triplicate. We observed the presence of NETs (red arrows), MPO (yellow arrows), and amoebic debris (asterisks). Additionally, the overlay of images (Merged Panels d, i, n, and s), and the brightfield images are observed (Panels e, j, o, t). Bar 20 μm.

During interactions of neutrophils and amoebae preincubated with GlcNAc at a concentration of 50 mM, the presence of DNA (Figures 4b, 4g, 4l, and 4q; red arrows) and MPO (Figures 4c, 4h, 4m, and 4r; yellow arrows) was observed, indicating the NETs formation by mouse neutrophils that interacted with amoebae (Figure 4d,i; blue arrows) and upon contact with amoebic debris (Figure 4n,s). Regarding the colocalization of NETs and MPO on trophozoites preincubated with GlcNAc carbohydrate, the decrease in DNA shows lower colocalization with a correlation that does not exceed 0.29, whereas the colocalization of the enzyme remains between 0.6 and 0.5 during all interaction times (Figure S4).

### 3.5. NETs Concentrations Decreased Significantly During Interactions Between Neutrophils and *E*. *histolytica* Preincubated With Carbohydrates GalNAc or GlcNAc

**Figure 5:**
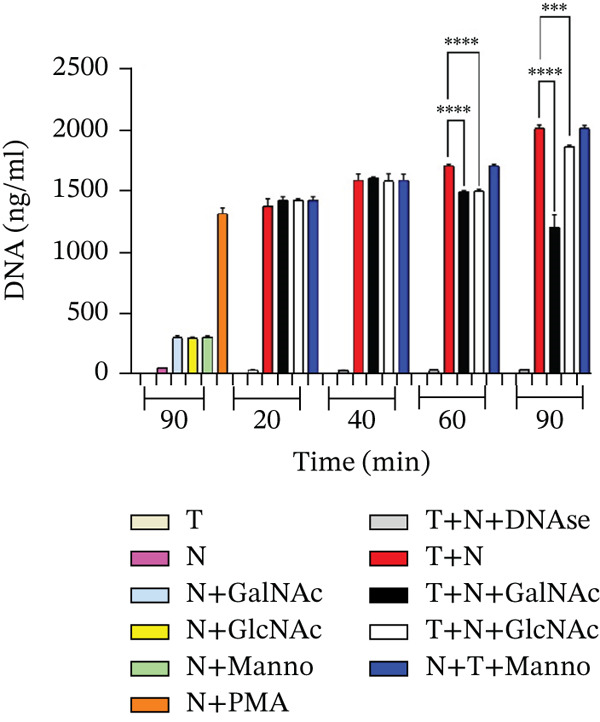
Quantification of DNA in the interactions of mouse neutrophils and *E*. *histolytica* preincubated with carbohydrates (50 mM). We used PMA (20 nmol/L) as a positive control and DNAse (4 U/mL) as a negative control. The data represent the mean ± SD (triplicate). *p* values were determined by two‐way ANOVA ( ^∗∗∗∗^ *p* < 0.0001;  ^∗∗∗^ *p* < 0.001) compared with the control group. N = neutrophil, T = trophozoites.

We quantified the concentration of DNA present in the NETs at 20, 40, 60, and 90 min of interaction in the supernatant of the interactions of neutrophils and trophozoites preincubated with carbohydrates at 50 mM (Figure 5). We observed a statistically significant decrease in the DNA concentration in the interactions of neutrophils and amoebae preincubated with GalNAc or GlcNAc compared with the concentration in the control group (*E. histolytica–neutrophil)* at 60 and 90 min. Furthermore, we observed a greater decrease in the DNA concentration in the interactions of neutrophils and amoebae preincubated with GalNAc at 90 min than in the control group. In the case of amoebae preincubated with Manno, we did not observe any significant difference compared with the control.

### 3.6. MPO Enzyme Activity Increases During Interactions Between Neutrophils and *E. histolytica* Preincubated With GalNAc or GlcNAc Carbohydrates

**Figure 6:**
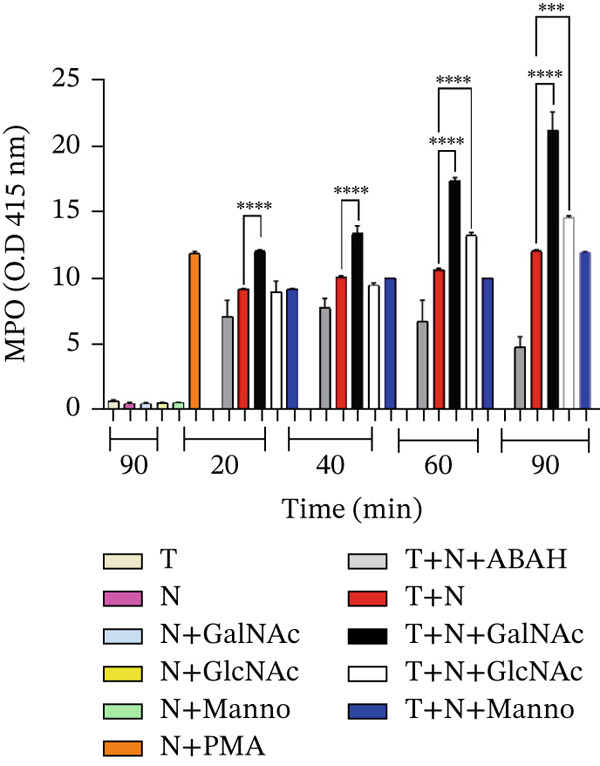
MPO enzyme activity in the interactions of neutrophils and *E*. *histolytica* preincubated with carbohydrates (GalNAc, GlcNAc, or Manno [50 mM]) measured by TMB assay. We used PMA as a positive control and ABAH as a negative control. The data represent the mean ± SD (triplicate). *p* values were determined by two‐way ANOVA ( ^∗∗∗∗^ *p* < 0.0001;  ^∗∗∗^ *p* < 0.001) compared with the control group and between groups. N = neutrophil, T = trophozoites.

From the supernatants obtained from the interactions of neutrophils and *E. histolytica* preincubated with 50 mM GalNAc, GlcNAc, or Manno at 20, 40, 60, and 90 min, the activity of MPO was quantified (Figure 6). We observed a significant increase in MPO enzyme activity in the interactions of neutrophils and amoebae preincubated with GalNAc from 20 to 90 min with respect to the interactions without preincubated amoebae, and the greatest increase in MPO activity was observed with *E. histolytica* preincubated with 50‐mM GalNAc at 90 min. We observed an increase in MPO enzyme activity in the interactions of neutrophils and *E. histolytica* preincubated with GlcNAc only at 60 and 90 min compared with the interactions without preincubated amoebae. We did not observe a statistically significant difference in the interactions of neutrophils and *E. histolytica* preincubated with Manno compared with the control.

### 3.7. Rosette Formation Decreases in the Interactions of Neutrophils and *E*. *histolytica* Preincubated With GalNAc or GlcNAc Carbohydrates

**Figure 7:**
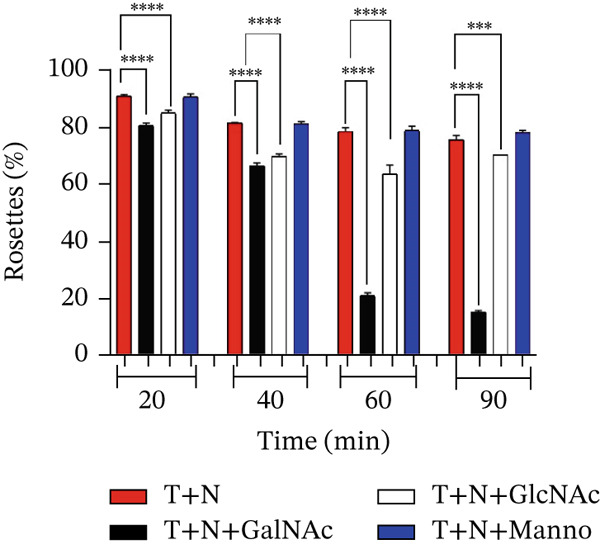
Assays of adherence of neutrophils to *E. histolytica* preincubated with carbohydrates. Adherence counts in the interactions of mouse neutrophils and *E. histolytica* trophozoites preincubated with or without GalNAc, GlcNAc, or Manno (50 mM). The data represent the mean ± SD (triplicate). *p* values were determined by two‐way ANOVA ( ^∗∗∗∗^ *p* < 0.0001;  ^∗∗∗^ *p* < 0.001) compared with the control group and between groups. N = neutrophil, T = trophozoites.

When we examined the adherence of trophozoites to mouse neutrophils, we counted those trophozoites that formed rosettes when they adhered to neutrophils after the incubation in the interactions of neutrophils and *E. histolytica* preincubated with 50‐mM GalNAc or GlcNAc (Figure 7). A statistically significant decrease in the adhesion of trophozoites preincubated with GalNAc to neutrophils (approximately 83%) was observed at 20 min, and 86.24% of the trophozoites preincubated with GlcNAc formed rosettes. After 40 min, a decrease in rosette formation of less than 66% with amoebae preincubated with GalNAc and 77% with amoebae preincubated with GlcNAc occurred. At 60 min of interaction, we observed rosette formation levels of only 20% with amoebae preincubated with GalNAc and 73% with amoebae preincubated with GlcNAc. At 90 min, we were able to observe rosette formation levels of 15% with amoebae preincubated with GalNAc and 71% with amoebae preincubated with GlcNAc. Finally, in the interactions of amoebae in the absence of carbohydrates or with Manno, no significant differences were observed.

## 4. Discussion

NETs are composed of DNA and MPO enzyme, as reported previously [10, 15]. NETs and MPO enzyme are important for damaging different types of pathogens. Ventura‐Juarez et al. demonstrated that human neutrophils release NETs upon in in vitro contact with *E. histolytica* trophozoites, producing a significant amoebicidal effect [15]. Contis et al. also reported that interactions of mouse neutrophils with *E. histolytica* trophozoites in the presence of DNAse or ABAH significantly reduced the presence of NETs and MPO enzyme; moreover, the viability of trophozoites increased [19]. Caro et al. reported the presence of NETs and MPO enzyme in *Besnoitia besnoiti* in interactions with bovine neutrophils for 1 h, which promoted parasite damage [24]. Abdallah et al. reported that damage to *T. gondii* is mediated by the formation of NETs after 4 h of incubation with C57BL/6 mouse neutrophils and that the formation of NETs and the release of MPO enzyme are crucial for parasite destruction [14]; these previous reports confirmed the important role of NETs in pathogen damage. To evaluate the presence of NETs against *E. histolytica*, we performed an immunofluorescence assay. First, in the interactions of neutrophils and amoebae without carbohydrates preincubation, we observed the presence of NETs and MPO enzyme colocalized in trophozoites (see colocalization analysis of fluorescence images in supporting information), although a part of NETs are dispersed around the trophozoites, they interact with amoeba. NETs caused damage over time because the traps were in close contact with *E. histolytica* trophozoites (20 and 40 min) and amoebic debris (60 and 90 min), similar to the NETS and MPO enzyme observed by Ventura‐Juarez et al., in which the authors reported that human neutrophils released NETs and MPO enzyme upon direct contact with *E. histolytica* trophozoites from 15 to 60 min of interaction [15]. Contis et al. also demonstrated the presence of NETs and MPO enzyme in mouse neutrophils and reported very significant increases in NETs and MPO enzyme compared with those in hamster neutrophils that interacted with trophozoites at 20, 40, 60, and 90 min [19]. In the present work, we observed trophozoites damage in the presence of MPO enzyme. In the interactions of neutrophils and *E*. *histolytica* preincubated with Manno, NETs, and MPO were very similar to the interactions of neutrophils and trophozoites not preincubated with carbohydrates. Manno is a carbohydrate unrelated to the recognition of amoebic lectins, and this result indicates that there is indeed no relationship in the adhesion of this carbohydrate. With respect to the interactions of neutrophils and *E*. *histolytica* preincubated with GalNAc or GlcNAc, we observed that NETs and the MPO enzyme partially colocalize with *E*. *histolytica* trophozoites, with correlation values ranging from 0.6 for interactions without sugar to correlations of 0.2 in interactions with GalNAc or GlcNAc, whereas MPO remains visible and with interactions that correlate from 0.7 for groups without sugar to 0.6 for interactions with trophozoites incubated with sugars. With respect to the carbohydrate–NETs relationship, *Aspergillus fumigatus*, a filamentous fungus that produces galactosaminoglycans (GAG), which are composed of galactose and GalNAc, has been reported to have no effect on NETs in neutrophils cocultured with *A. fumigatus* strains, and NET formation is correlated with differences in the GAG‐dependent susceptibility of strains [25]. Our results revealed that, in the interactions of neutrophils and *E*. *histolytica* preincubated with GalNAc or GlcNAC, the decreased release of NETs is likely due to the inhibition of adhesion of *E. histolytica* to neutrophils caused by the blocking of amoebic lectins by the previous recognition of these carbohydrates, which favors other effector mechanisms of the neutrophil, such as the release of MPO enzyme to damage the parasite. To corroborate this possibility, we quantified the extracellular DNA found in the NETS in the interactions and observed significant decrease in DNA levels at 60 and 90 min in the interaction of neutrophils and *E*. *histolytica* preincubated with GalNAc. Additionally, in the interactions of neutrophils and *E*. *histolytica* preincubated with GlcNAc, a decrease in DNA was observed compared with that in the control group of neutrophils and *E*. *histolytica* without carbohydrates preincubation or in amoebae preincubated with Manno, a carbohydrate unrelated with amoebic adhesion. Unlike in our work, it has been reported that the interaction of *Porphyromonas gingivalis* with human neutrophils in the presence of 25‐mM glucose for 3 h resulted in an increase in the release of NETs, but this stimulus was lower when the neutrophils alone were incubated without glucose stimulus [26]. In our work, we preincubated trophozoites with GalNAc, GlcNAc, or Manno at 50 mM before the interaction of amoebae with neutrophils, and we did not observe significant stimulation with GalNAc or GlcNAc in the formation of NETs compared with the other working groups.

MPO enzyme activity could also participate in damage to *E. histolytica* when it is released from neutrophils; therefore, we quantified this activity. As shown in Figure 6, we observed a significant increase in MPO enzyme activity during the interaction of neutrophils with *E*. *histolytica* preincubated with GalNAc. An increase in MPO enzyme activity was observed at all interaction times. The greatest increase in MPO enzyme activity was observed in the interaction of neutrophils with *E*. *histolytica* preincubated with GalNAc at 90 min. In terms of the interactions of neutrophils and *E*. *histolytica* preincubated with GlcNAc, an increase in MPO enzyme activity was observed only at 60 and 90 min of interaction compared with the group of trophozoites without carbohydrate preincubation. Our results suggest that the damage caused to trophozoites could be the result of the MPO enzyme activity stimulated mainly by the blockade of amoebic lectins with the carbohydrate GalNAc. This increase in MPO enzyme activity has been previously demonstrated in a murine model of ALA resistance (BALB/c mice), where the lesion is resolved in the acute phase of the infection, where neutrophils participate significantly in the resolution of the damage and where increased MPO enzyme activity and elevated MPO gene expression are observed, which is correlated with the resolution of damage and the participation of the MPO enzyme in the damage of *E. histolytica* trophozoites [27]. Flores‐Huerta et al. reported an increase in MPO enzyme activity in the neutrophils of mice in response to *Naegleria fowleri*, with the greatest activity reported at 120 min [28]. Compared with that in the control group, the MPO enzyme activity was increased in the interactions of neutrophils with *E*. *histolytica* preincubated with mainly GalNAc. We believe that the presence of GalNAc stimulates neutrophils to release MPO enzyme in short amounts of time when the adhesion is blocked.

To prove that lectins prevent adhesion between amoeba and neutrophil to induce neutrophil activation and consequently damage *E. histolytica*, we carried out adhesion blocking assays to measure rosette formation. Our study revealed a significant reduction in rosette formation during interactions between neutrophils and *E*. *histolytica* preincubated with 50‐mM GalNAc at all tested times. Previously, the presence of 50‐mM GalNAc was reported to inhibit amoebic adherence, prevent neutrophil death, and allow neutrophils to destroy the amoeba [6, 8, 29]. GalNAc significantly blocked the adhesion of amoebae to neutrophils, inhibited NETs formation, and increased MPO enzyme activity. These results suggest that the inhibition of the amoebic 260‐kDa lectin inhibited by Gal/GalNAc likely allows the MPO enzyme of the neutrophil to be properly activated and consequently destroy the amoebae.

The importance of the MPO has been described in Balb/c mice because it favors the positive resolution of amoebic liver damage due to the amoebicidal effect of MPO [27]. MPO plays a key role in the resolution of *E. histolytica* infection in a resistant model compared with a susceptible model (the hamster), where MPO activity is lower, which could indicate the key role of this enzyme against *E. histolytica* [19]. Our study revealed that other neutrophil mechanisms could be involved in the damage of *E. histolytica* trophozoites, most likely through the direct action of the MPO enzyme. Future studies should be performed to determine the specific involvement of amoebic lectins in blocking neutrophil activation and the involvement of various neutrophil enzymes in amoebic damage.

## 5. Conclusion

Our data revealed that mainly GalNAc carbohydrate prevented *E. histolytica* adhesion to neutrophils, suggesting that the involvement of the amoebic Gal/GalNAc lectin in this event also reduced the release of NETs. This decrease in NETs production in the interactions between neutrophils and amoeba preincubated with GalNAc did not prevent trophozoite damage, suggesting that the activity of other mechanisms other that of neutrophils is involved in amoebic damage, such as that of MPO enzyme, which increases in the interactions of neutrophils and amoebae preincubated with GalNAc, probably through the inhibition of the amoebic Gal/GalNAc lectin. MPO enzyme activity could be mainly responsible for the damage of *E. histolytica*.

## Funding

Participants received any of the following supports: COFAA‐IPN and EDI‐IPN.

## Conflicts of Interest

The authors declare no conflicts of interest.

## Supporting Information

Additional supporting information can be found online in the Supporting Information section.

## Supporting information


**Supporting Information 1** Figure S1: Colocalization analysis of fluorescence images in neutrophil–amoeba interactions without preincubation with carbohydrates. The presence of NETs and the MPO enzyme in the interactions of mouse neutrophils and *E. histolytica* was evaluated at 20, 40, 60, and 90 min. The images were superimposed to determine the correlation of interaction DNA and MPO. The size of ROIs was 336 × 336 pixels. The scale bar represents 20 *μ*m. The *x*‐axis denotes trophozoite labels′ intensity, whereas the *y*‐axis shows DNA or MPO intensity colocalization. The R total was calculated by Fiji.

**Supporting Information 2** Figure S2: Colocalization analysis of fluorescence images in interactions of neutrophils with amoebae preincubated with mannose. The presence of NETs and the MPO enzyme in the interactions of mouse neutrophils and *E. histolytica* was evaluated at 20, 40, 60, and 90 min. The images were superimposed to determine the correlation of interaction DNA and MPO. The size of ROIs was 336 × 336 pixels. The scale bar represents 20 *μ*m. The *x*‐axis denotes trophozoite labels′ intensity, whereas the *y*‐axis shows DNA or MPO intensity colocalization. The R total was calculated by Fiji.

**Supporting Information 3** Figure S3: Colocalization analysis of fluorescence images in interactions of neutrophils with amoebae preincubated with GalNAc. The presence of NETs and the MPO enzyme in the interactions of mouse neutrophils and *E. histolytica* was evaluated at 20, 40, 60, and 90 min. The images were superimposed to determine the correlation of interaction DNA and MPO. The size of ROIs was 336 × 336 pixels. The scale bar represents 20 *μ*m. The *x*‐axis denotes trophozoite labels′ intensity, whereas the *y*‐axis shows DNA or MPO intensity colocalization. The R total was calculated by Fiji.

**Supporting Information 4** Figure S4: Colocalization analysis of fluorescence images in interactions of neutrophils and amoebae preincubated with GlcNAc. The presence of NETs and the MPO enzyme in the interactions of mouse neutrophils and *E. histolytica* was evaluated at 20, 40, 60, and 90 min. The images were superimposed to determine the correlation of interaction DNA and MPO. The size of ROIs was 336 × 336 pixels. The scale bar represents 20 *μ*m. The *x*‐axis denotes trophozoite labels′ intensity, whereas the *y*‐axis shows DNA or MPO intensity colocalization. The R total was calculated by Fiji.

## Data Availability

The data that support the findings of this study are available from the corresponding author upon reasonable request.
