# Supplementary figures and images for: GalNAc Carbohydrate Prevents the Formation of Neutrophil Extracellular Traps and Increase Myeloperoxidase Enzyme Activity in Interactions of Neutrophils and Entamoeba histolytica Preincubated With GalNAc
Source: Biomed Res Int. 2026 Apr 10;2026:8280585. doi: 10.1155/bmri/8280585 (PMC13067049; doi:10.1155/bmri/8280585)

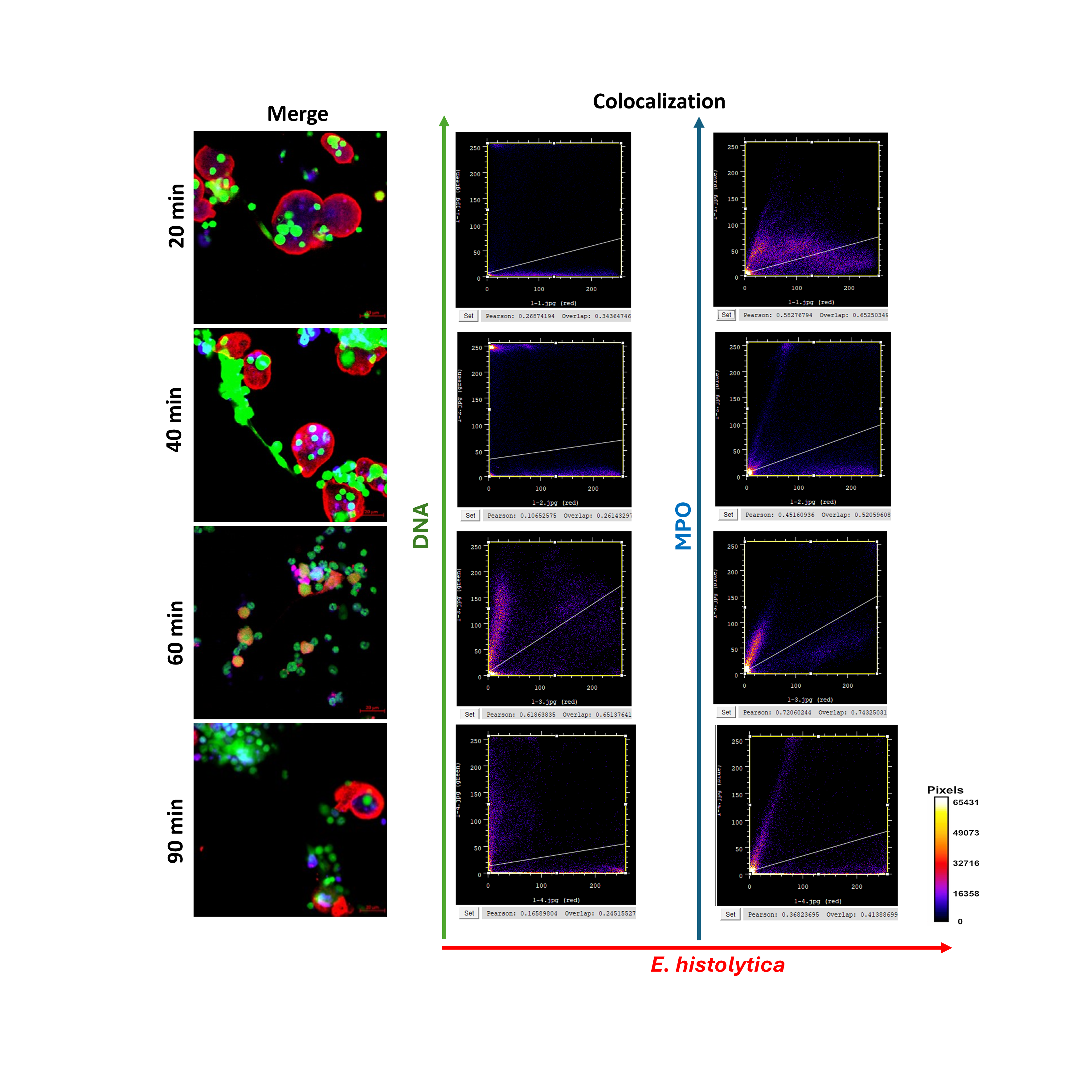

Supplement: Supplementary file 1 — Supporting Information 1 Figure S1: Colocalization analysis of fluorescence images in neutrophil–amoeba interactions without preincubation with carbohydrates. The presence of NETs and the MPO enzyme in the interactions of mouse neutrophils and E. histolytica was evaluated at 20, 40, 60, and 90 min. The images were superimposed to determine the correlation of interaction DNA and MPO. The size of ROIs was 336 × 336 pixels. The scale bar represents 20 μm. The x‐axis denotes trophozoite labels′ intensity, whereas the y‐axis shows DNA or MPO intensity colocalization. The R total was calculated by Fiji. [file BMRI-2026-8280585-s003.png]

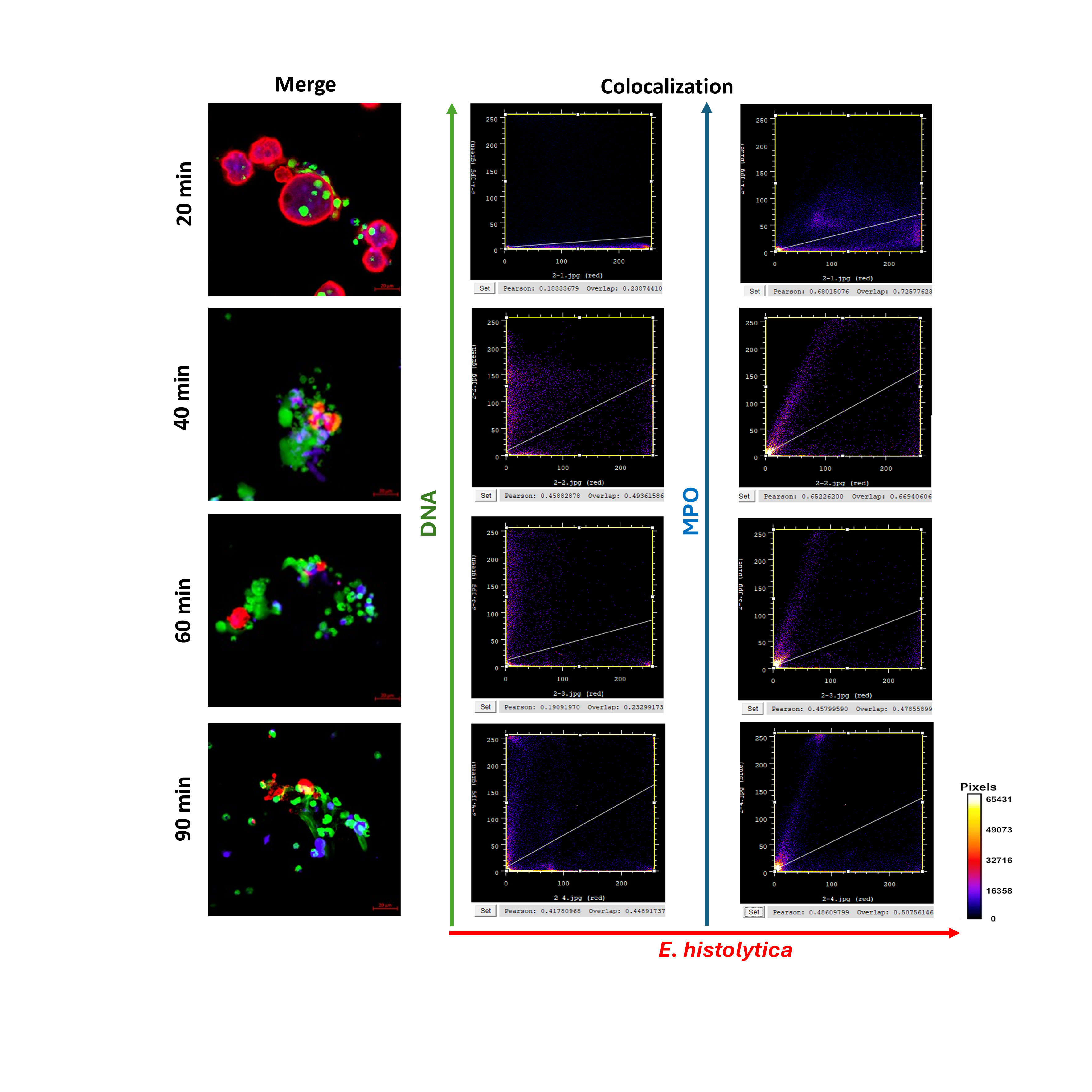

Supplement: Supplementary file 2 — Supporting Information 2 Figure S2: Colocalization analysis of fluorescence images in interactions of neutrophils with amoebae preincubated with mannose. The presence of NETs and the MPO enzyme in the interactions of mouse neutrophils and E. histolytica was evaluated at 20, 40, 60, and 90 min. The images were superimposed to determine the correlation of interaction DNA and MPO. The size of ROIs was 336 × 336 pixels. The scale bar represents 20 μm. The x‐axis denotes trophozoite labels′ intensity, whereas the y‐axis shows DNA or MPO intensity colocalization. The R total was calculated by Fiji. [file BMRI-2026-8280585-s004.png]

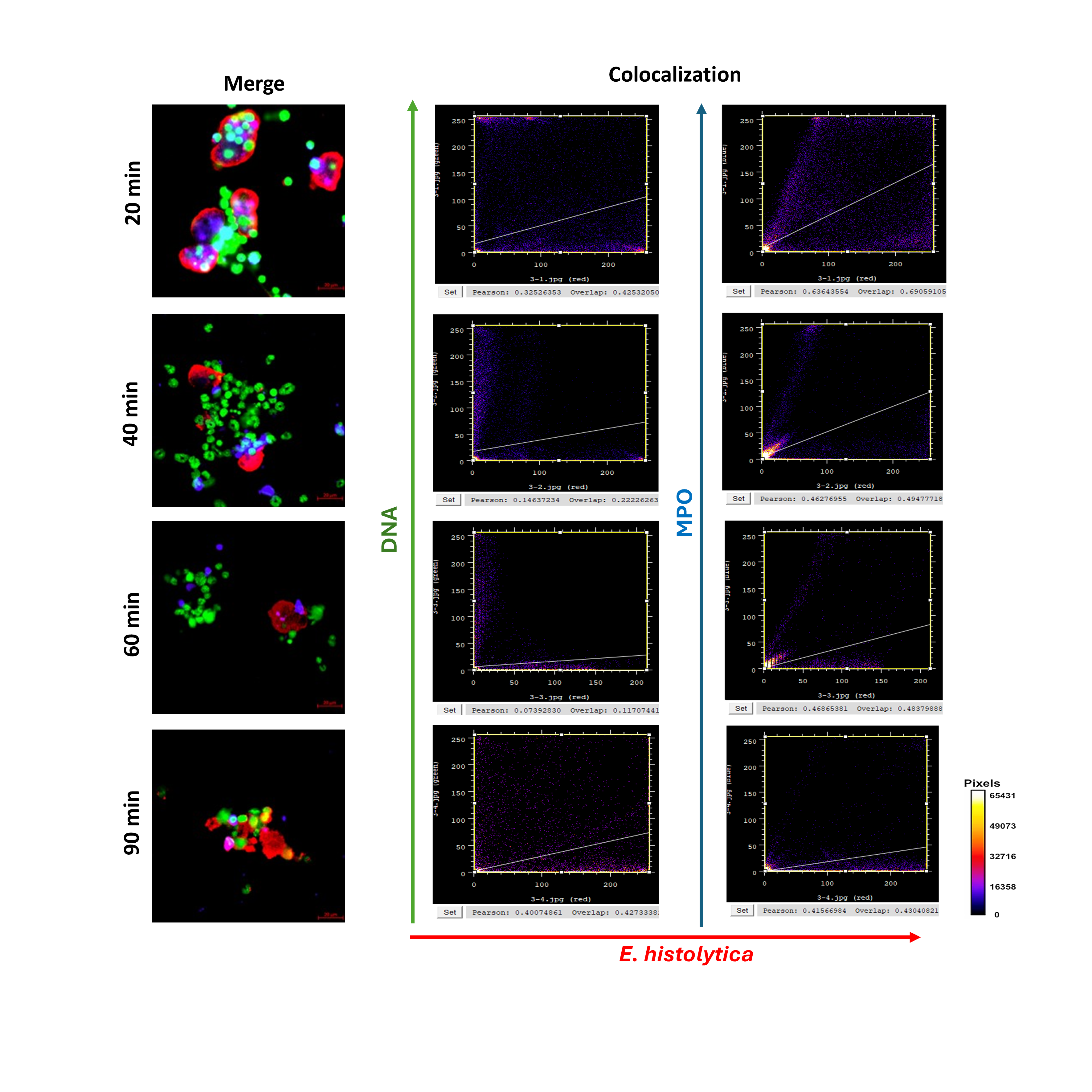

Supplement: Supplementary file 3 — Supporting Information 3 Figure S3: Colocalization analysis of fluorescence images in interactions of neutrophils with amoebae preincubated with GalNAc. The presence of NETs and the MPO enzyme in the interactions of mouse neutrophils and E. histolytica was evaluated at 20, 40, 60, and 90 min. The images were superimposed to determine the correlation of interaction DNA and MPO. The size of ROIs was 336 × 336 pixels. The scale bar represents 20 μm. The x‐axis denotes trophozoite labels′ intensity, whereas the y‐axis shows DNA or MPO intensity colocalization. The R total was calculated by Fiji. [file BMRI-2026-8280585-s002.png]

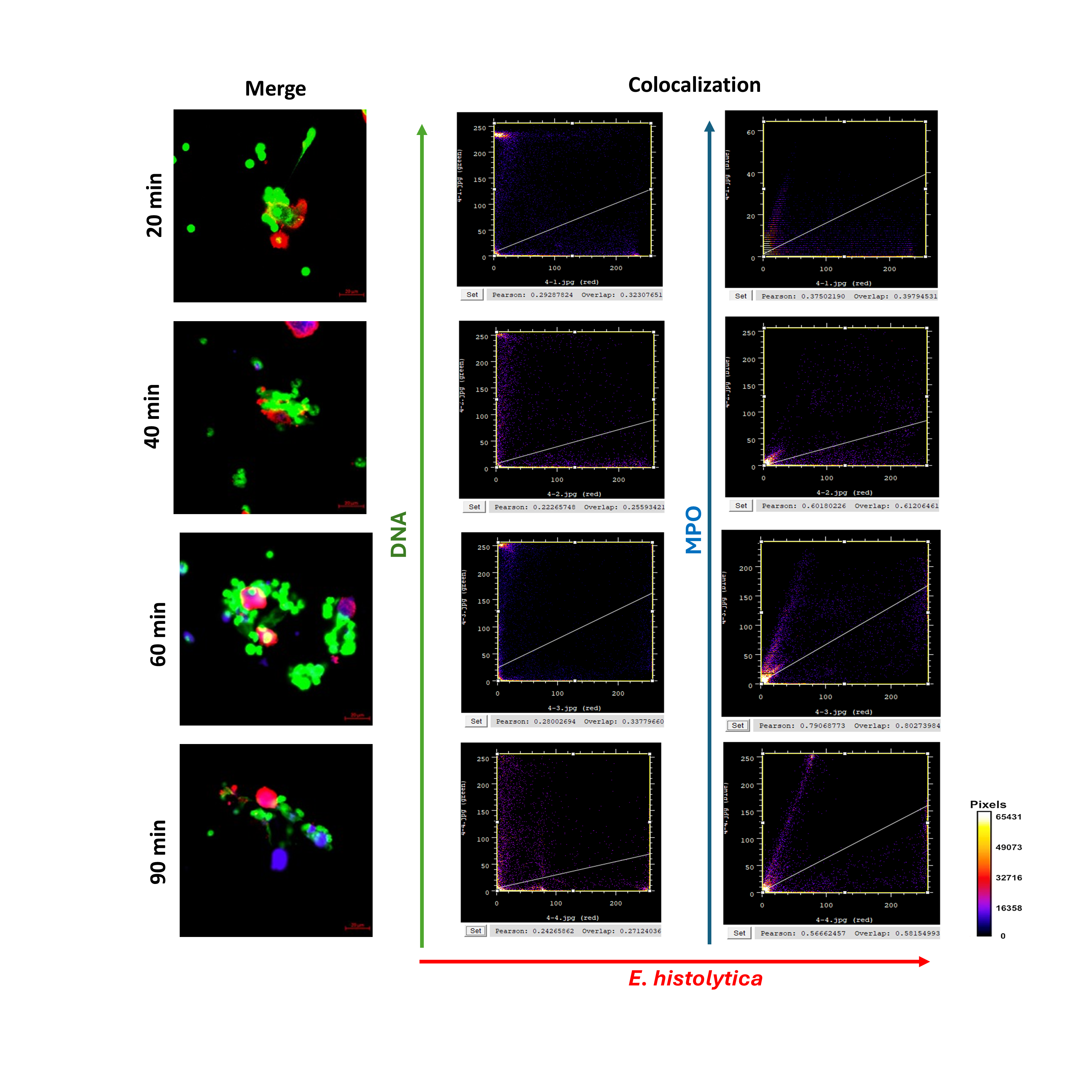

Supplement: Supplementary file 4 — Supporting Information 4 Figure S4: Colocalization analysis of fluorescence images in interactions of neutrophils and amoebae preincubated with GlcNAc. The presence of NETs and the MPO enzyme in the interactions of mouse neutrophils and E. histolytica was evaluated at 20, 40, 60, and 90 min. The images were superimposed to determine the correlation of interaction DNA and MPO. The size of ROIs was 336 × 336 pixels. The scale bar represents 20 μm. The x‐axis denotes trophozoite labels′ intensity, whereas the y‐axis shows DNA or MPO intensity colocalization. The R total was calculated by Fiji. [file BMRI-2026-8280585-s001.png]
